# Molecular characteristics of segment 5, a unique fragment encoding two partially overlapping ORFs in the genome of rice black-streaked dwarf virus

**DOI:** 10.1371/journal.pone.0224569

**Published:** 2019-11-07

**Authors:** Hongyue Zu, Hong Zhang, Minhao Yao, Jiayue Zhang, Hong Di, Lin Zhang, Ling Dong, Zhenhua Wang, Yu Zhou

**Affiliations:** Key Laboratory of Germplasm Enhancement, Physiology and Ecology of Food Crops in Cold Region, Northeast Agricultural University, Changjiang Road, Xiangfang District, Harbin, Heilongjiang Province, China; Oklahoma State University, UNITED STATES

## Abstract

Rice black-streaked dwarf virus (RBSDV), a ds-RNA virus in *Fijivirus* genus with family *Reoviridae*, which is transmitted by the small brown planthopper, is responsible for incidence of maize rough dwarf disease (MRDD) and rice black-streaked dwarf disease (RBSDD). To understand the variation and evolution of S5, a unique fragment in the genome of RBSDV which encodes two partially overlapping ORFs (ORF5-1 and ORF5-2), we analyzed 127 sequences from maize and rice exhibiting symptoms of dwarfism. The nucleotide diversity of both ORF5-1 (π = 0.039) and ORF5-2 (π = 0.027) was higher than that of the overlapping region (π = 0.011) (*P* < 0.05). ORF5-2 was under the greatest selection pressure based on codon bias analysis, and its activation was possibly influenced by the overlapping region. The recombinant fragments of three recombinant events (14NM23, 14BM20, and 14NM17) cross the overlapping region. Based on neighbor-joining tree analysis, the overlapping region could represent the evolutionary basis of the full-length S5, which was classified into three main groups. RBSDV populations were expanding and haplotype diversity resulted mainly from the overlapping region. The genetic differentiation of combinations (T127-B35, T127-J34, A58-B35, A58-J34, and B35-J34) reached significant or extremely significant levels. Gene flow was most frequent between subpopulations A58 and B35, with the smallest |*Fst*| (0.02930). We investigated interactions between 13 RBSDV proteins by two-hybrid screening assays and identified interactions between P5-1/P6, P6/P9-1, and P3/P6. We also observed self-interactive effects of P3, P6, P7-1, and P10. In short, we have proven that RBSDV populations were expanding and the overlapping region plays an important role in the genetic variation and evolution of RBSDV S5. Our results enable ongoing research into the evolutionary history of RBSDV-S5 with two partly overlapping ORFs.

## Introduction

Rice black-streaked dwarf virus (RBSDV; genus *Fijivirus*, family *Reoviridae*), transmitted by the small brown planthopper (SBPH; Laodelphax striatellus Fallén), causes serious maize and rice losses worldwide, particularly in China [1,2]. Previous studies demonstrated the unique RBSDV S5 among ten ds-RNA segments with two partially overlapping ORFs but in a different reading frame [3]. RBSDV encodes 13 proteins that have been partially determined on their functions and interaction among proteins in rice and maize [2,3].

RBSDV genome segment S5 (S5) contains a known major ORF and second partially-overlapping ORF in a different reading frame. Segment S5 belongs to functionally bicistronic structures of viruses in infected plants [4], and codes the structural protein (SP) P5-1 and the non-structural protein (NSP) P5-2. RBSDV P5-1 is an established component of viroplasms, which also include P9-1 and P6 [2,4]. Southern rice black-streaked dwarf virus (SRBSDV) P5-1 is involved in formation of viroplasms by interacting with P6 in infected cells [5]. RBSDV P5-2 was localized in the chloroplasts in RBSDV-infected plant cells [6], though its function is yet unknown. Both P5-1 and P5-2 show traits indicating that they may play a role in viral replication, but as of yet the codon usage bias of their encoding dsRNAs and overlapping regions as well as their genetic structure have not been described.

Virus populations break the equilibrium by competing favorably with environment which responds to even small changes in the corresponding environment and drive virus evolution [7]. It is clear that studying the genetic variation and evolution of plant viruses proposes an important strategy for agricultural production due to the warming climate and the harsh ecosystem [8–10]. Evolution analysis of RNA viruses was achieved by codon usage bias, phylogenetic tree, genetic distance, selection pressure, genetic differentiation and gene flow which have been examined in some ss-RNA viruses [11,12] and ds-RNA viruses [4,13]. However, evolutionary analysis has only been conducted in a few viruses with overlapping open reading frames (ORFs).

Overlapping ORFs are fairly common in viruses [14], including Hepatitis B virus (HBV, ds-DNA viruses) [15], cucumber mosaic virus (CMV, ss-RNA viruses) [16], Rosellinia necatrix megabirnavirus 1 (RnMBV1, ds-RNA viruses) [17], and Rice black-streaked dwarf virus (RBSDV, ds-RNA viruses) [4]. Many ds-RNA viruses, particularly those with three or fewer genome segments, have the ability to translate a single mRNA segment into multiple proteins, which can play different roles in virus reproduction and pathogenesis [10]. For example, CMV contains partially overlapping ORFs 2a and 2b [16], and different pathogenicity was mediated by the 2b proteins, rather than the C-terminal overlapping parts of the 2a proteins [18].

Previous research has focused on understanding the mechanisms that drive the evolutionary history and geographic dispersion of plant viruses. Understanding their genetic structure and diversity has implications for increased knowledge of emergence, dispersion, and pathogenicity [19]. However, analysis of codon usage and genetic structure has not been previously conducted for the S5 segment of RBSDV with two partly overlapping ORFs. In the current study, we examine the codon usage bias and patterns of genetic structure of RBSDV S5 from 127 isolates with symptoms of maize rough dwarf disease (MRDD) and rice black-streaked dwarf disease (RBSDD) from eight geographic locations. Our findings enable better understanding of the evolutionary history and geographic dispersion of the S5 ds-RNA segment of RBSDV with two partly overlapping ORFs.

## Materials and methods

### Sources of virus samples

A total of 127 maize or rice samples exhibiting dwarfism symptoms were collected from eight different regions (I: Beijing; II: Tangshan, Hebei Province; III: Baoding, Hebei Province; IV: Jinan, Shandong Province; V: Jining, Shandong Province; VI: Zhengzhou, Henan Province; VII: Yancheng, Jiangsu Province; VIII: Nanjing, and Jiangsu Province) in 2013 and 2014. Rice samples from four locations were collected together with Dr. Jie Shi and Dr. Bo Li of HAAFS (Hebei Academy of Agriculture and Forestry Sciences, III), Zhao-Wen Sun of JARI (Jining Agricultural Reseach Institutes, V), Dr. Shuang-Gui Tie and Dr. Xiao-Hua Han of HAAS (Henan Academy of Agricultural Sciences, VI), and Dr. Yan-Ping Chen of JAAS (Jiangsu Academy of Agricultural Sciences, VIII) respectively. In addition to the above four locations, maize samples were also collected together with Yu-Zhou of CAAS (Chinese Academy of Agricultural Sciences, I), Wen-Yue Tong of TARI (Tangshan Agricultural Reseach Institutes, II), and Dr. Zhao-Dong Meng and Dr. Qi Sun from SAAS (Shandong Academy of Agricultural Sciences, IV). Our research was not conducted on private land, and we confirmed that no endangered or protected species were involved in our research. The work of collecting samples in the experimental fields of every academy of agricultural sciences was carried out with professional researchers of local institutions, which did not require special permission.

The 127 maize or rice plants were designated T127 and divided into three subpopulations (**S1 Table**). Subpopulation A58 is composed of 58 maize or rice plant samples, among which the virus-infected maize plants from A58 included one from Beijing (I), three from Tangshan (II), eight from Baoding (III), seven from Jinan (VI), six from Jining (V), six from Zhengzhou (VI), two from Yancheng (VII), and five from Nanjing (VIII). Rice plants from A58 were also collected near the same locations in which maize was cultivated, including two from Baoding (III), five from Jining (V), ten from Zhengzhou (VI), and three from Nanjing (VIII). Subpopulation B35 is composed of 35 dwarf maize plants collected from Beijing (I) in 2014, and subpopulation J34 is composed of 34 dwarf maize plants collected from Jining (V) in 2014. Leaf samples from infected plants were immediately frozen in liquid nitrogen and stored at -80°C until used for analyses. The GenBank accession numbers of these 127 sequences were from MH999291 to MH999417.

### RNA extraction, RT-PCR, and sequencing

RBSDV ds-RNA was extracted from above-mentioned rice and maize samples. Synthesis of first-strand cDNA and PCR amplification of RBSDV S5 were performed using a Fast Quant RT Kit (TIANGEN, Beijing, China) and KOD-Plus-Neo enzyme (TOYOBO, Osaka, Japan) with three pairs of S5 specific primers. The primers used to amplify the S5 segment were S5-F-1/S5-1-1, S5-R-1/S5-2-1 and S5-3-1/S5-4-1 (**S2 Table**). PCR products were sequenced using the dideoxy chain-termination method (The AuGCT DNA-SYN Biotechnology Company, Beijing, China). At least three independent replicates of the PCR reactions of each collected sample were conducted to obtain accurate sequences. DNAMAN software and Jemboss1.5 (EMBOSS, Cambridge, UK) were used for sequence assembly and analysis [20].

### Sequence variants and nucleotide diversity

Nucleotide sequences were aligned using MegAlign program in DNAStar5.01 with default settings (Madison, WI, USA). Sequences were subsequently manually adjusted for the ORFs [21,22]. We analyzed nucleotide diversity (π) in the S5 sequences with the sliding-window method using a 200-bp window in 100-bp steps and a 20-bp window in 10-bp steps in TASSEL 3.0 [23]. We used this method to analyze ORF5-1, ORF5-2, and OR of A58, B35, J34, and T127, respectively.

### Codon usage bias analysis of the S5 sequences

Codon usage in ORF5-1, ORF5-2, OR5-1 and OR5-2 of A58, B35, J34, and T127 was assessed using Codon W 1.4.4 (http://sourceforge.net/projects/codonw/), based on the values of codon adaptation index (*CAI*), The codon bias index (*CBI*), Effective number of codons (*Nc*), *GC3s*, and *GC*.

The codon adaptation index (*CAI*) was employed to analyze codon usage bias, or the codons preferred in highly expressed genes. *CAI* can vary from 0 to 1, with higher values indicating greater deviation from a reference set of genes and thus higher usage bias [24].

The codon bias index (*CBI*) was utilized for assessing the composition of preferred codons. A positive value for *CBI* indicates that mRNA triplets are constructed with preferred codons, while a negative value shows that non-preferred codons are used more often than expected, and a value of zero reveals that codons are used randomly.

Effective number of codons (*Nc*) is a measure of the state of codon usage bias in genes by measuring the nonuniformity of synonymous codon usage. Values of *Nc* range from 20, indicating extreme bias where only codon is exclusively used for each amino acid, to 61 indicating that the use of alternative synonymous codons is equally likely [24]. *Nc* plots (a plot of *Nc* versus *GC3s*, the frequency of (G + C) at the synonymous third positions of codons) can be used to examine patterns of codon usage across genes and organisms. Points lie on or below a theoretical curve (continuous curve between *Nc* and *GC3s*) if *GC3s* is the only determinant.

The *GC* content of the first, second, and third codon positions (*GC1*, *GC2*, and *GC3* respectively) were then calculated. SPSS 16.0 software (SPSS Inc., Chicago, IL, USA) was used to analyze the differences between A58, B35, J34, and T127 based on ORF5-1, ORF5-2, OR5-1, and OR5-2.

### Recombination and phylogenetic analyses of the S5 sequences

CLUSTAL W was used with the default settings to align amino acid and nucleotide sequences [25]. These sequences were then adjusted manually for the ORFs. The 3 SEQ, BOOTSCAN, CHIMAERA, GENECONV, Maximum Chi Square (MAXCHI), RDP, and Sister Scanning (SISCAN) programs in the RDP 4.22 software package were used to detect possible recombination sites in these S5 sequences, using the default settings with selection of ‘linear sequence’ and ‘disentangling overlapping signals’ [26]. Only sequences that were supported by at least six methods with a 100 simulated datasets at *P* < 0.05 were considered recombinant events. MEGA 7.0.18 software was used to construct phylogenetic trees for the S5 sequences with the neighbor-joining (NJ) method and 1000 bootstrap replicates [25]. Only those values with greater than 50% are reported.

### Assessment of selection pressure and population expansion

To estimate selection pressure imposed upon two partially overlapping ORFs in S5, the *Ka/Ks* ratio (number of nonsynonymous substitutions to synonymous substitutions) was calculated for each ORF5-1, ORF5-2, OR5-1 and OR5-2 by using DnaSP 5.0 software with 127 isolations [27]. Values greater than 1 indicate positive or Darwinian selection while less than 1 indicates purifying or stabilizing selection. A ratio of exactly 1 indicates neutral or no selection.

DnaSP 5.0 was also used to estimate Tajima’s D, Fu & Li’s D and F, and haplotype diversity [27]. Negative values for Tajima’s D or Fu & Li’s D or F indicate a low frequency of polymorphism in a population. Haplotype diversity represents the frequencies and numbers of haplotypes in a population, and ranges from 0 to 1.

### Measuring genetic differentiation and gene flow

DnaSP 5.0 software was used to measure genetic differentiation and gene flow between subpopulations [27]. The three permutation-based tests of Ks*, Z (rank statistic), and Snn (nearest-neighbor statistic) detected genetic differentiation. Ks* is calculated as the average number of differences between sequences regardless of geographic origin. Z is a weighted sum of Z1 and Z2, with Zi as the average rank of all dij.lk values for pairs of sequences from locality i. Snn measured how often the ‘nearest neighbors’ originated from the same locality. When Ks* and Z were *P* < 0.05, genetic differentiation was considered to have occurred.

Gene flow between subpopulations was estimated with *Nm* (the number of migrants) and *Fst* (the degree of genetic differentiation). If *Nm* < 1, reduced gene flow and increased genetic drift have resulted in local population differentiation [28]. *Fst* ranges from 0 to 1, representing undifferentiated and fully differentiated populations, respectively. *Fst* value of 0.33 can be considered a threshold, below which gene flow is frequent, and above which gene flow occurs infrequently [29, 30].

### Interaction between P5-1, P5-2, and other RBSDV proteins measured by two-hybrid screenings

Two-hybrid screenings (originally known as yeast two-hybrid system or Y2H) were performed following established methods with the Matchmaker® Gold Yeast Two-Hybrid System User Manual (Clontech, Kyoto, Japan). Co-transformants were mated and plated on SD-Leu-Trp media, and RBSDV-interacting proteins were selected on a SD-Trp-Leu-His-Ade/X-α-gal/AbA media (10 mg X-α-gal in 500μL DMF and 1mg Aureobasidin A in 2ml alcohol) at 30°C for 5 d. These yeasts which carry AD:prey and BD:bait grow and turn blue on SD-Trp-Leu-His-Ade/X-α-gal/AbA media when a positive protein interaction occurs, via activation and transcription of independent reporter genes. Yeasts co-transformed with pGBKT7-53/pGADT7-T were used as a positive control and yeasts co-transformed with pGBKT7-Lam/pGADT7-T were used as the negative control. For autoactivation assays, yeasts co-transformed with BD fused to the different RBSDV proteins and empty vector AD were plated on SD-Trp-Leu medium and transferred to SD-Trp-Leu-His-Ade/X-α-gal/AbA media, and the appearance of blue strain was monitored. Construction of recombinant plasmids was performed using the *pEASY*^®^-Ubi Seamless Cloning and Assembly Kit (TRAN, Beijing, China) (**S3 Table**), and 143 pairwise combinations consisting of BD fused to 11 RBSDV proteins (except P7-2 and P8 due to their autoactivation) and AD fused to 13 RBSDV proteins were co-transformed into Y2H strains. Interactions between RBSDV proteins were performed for at least three replicates and determined based on whether or not blue yeast strains appeared.

## Results

### Analysis of nucleotide sequence variation and diversity

**Fig 1:**
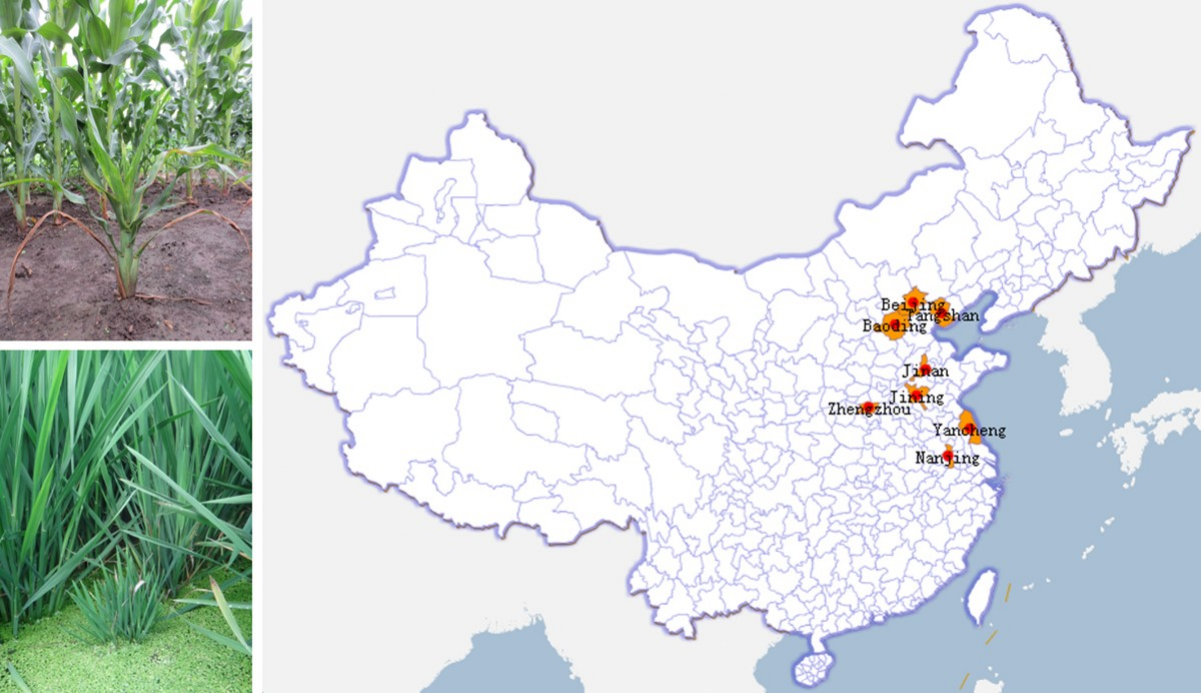
Plants of maize and rice with infected RBSDV and geographic distribution in China.

**Fig 2:**
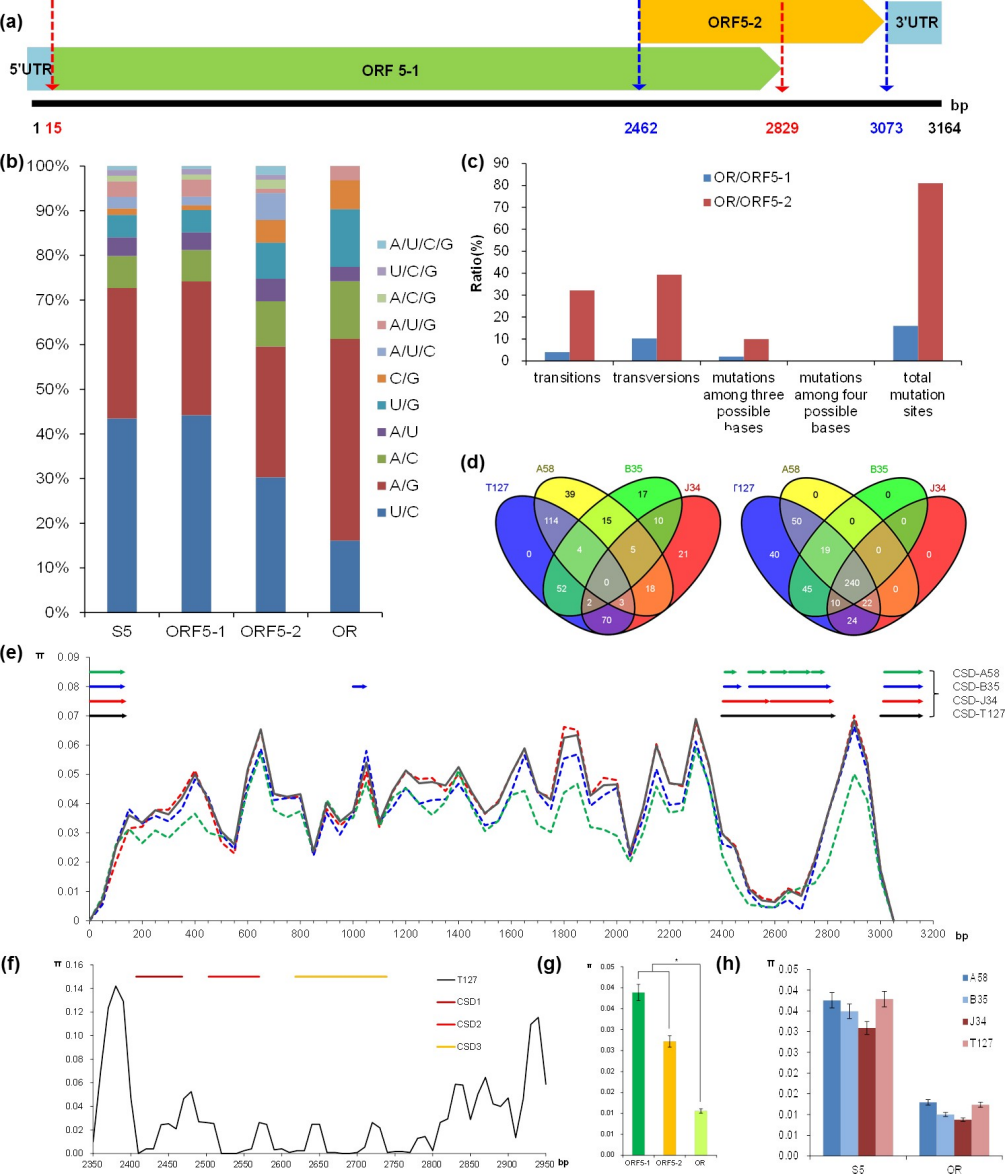
Analysis of nucleotide sequence variation and diversity in RBSDV-S5. (**a):** Obtaining of the open reading frames. (**b):** Analysis of nucleotide variation type in S5. **(c):** Analysis of rate of mutated bases between OR/ORF5-1 and OR/ORF5-2. **(d):** Venn analysis of singleton variable sites and parsimony-informative sites among populations. **(e):** Sliding-window analysis of nucleotide sequence diversity in complete S5 sequences calculated using a 100-bp window and 50-bp steps in subpopulation T127. (**f):** Sliding-window analysis of nucleotide sequence diversity in S5 from nt 2350 to nt 2950 calculated using a 20-bp window and 10-bp steps in subpopulation T127. **(g):** Significant difference analysis of nucleic acid diversity of S5 with 127 isolations based on ORF5-1, ORF5-2, and OR. (**h):** Significance analysis of nucleic acid diversity among populations.

The 127 plant samples exhibiting symptoms of RBSDV collected from eight locations(Fig 1) and divided into three subpopulations (A58, B35, J34 and A58+B35+J34 = T127) were analyzed for nucleotide sequence variation and diversity. Two open reading frames (ORF5-1 and ORF5-2) with overlapping regions (OR with 368 bp) are predicted in the S5 fragment (Fig 2A).

Across these 127 viral isolates, an average of one mutation site per four base pairs was detected among the ORF5-1, in total 627 nucleotide mutation sites, including 221 singleton and 406 parsimony-informative sites. Mutations included 465 transitions (U/C: 277; A/G: 188), 107 transversions (A/C: 44; A/U: 25; U/G: 31; C/G: 7), and 51 mutations across 3 possible bases (A/U/C: 12; A/U/G: 24; A/C/G: 7; U/C/G: 8) and 4 mutations among four possible bases (Fig 2B). An average of one nucleotide mutation per six base pairs was observed in ORF5-2, for a total of 99 nucleotide mutation sites, including 41 singleton and 58 parsimony-informative sites. These mutations included 59 transitions (U/C: 30; A/G: 29), 28 transversions (A/C: 10; A/U: 5; U/G: 8; C/G: 5), 10 mutations among three possible bases (A/U/C: 6; A/U/G: 1; A/C/G: 2; U/C/G: 1), and two mutations among four possible bases (Fig 2B). Neither ORF5-1 and ORF5-2 showed no insertions or deletions. In addition to the four-base mutations, the ratio of mutated bases in the OR region to total ORF5-2 mutations was significantly higher than that of mutated bases in the OR region to total ORF5-1 mutations, and the ratio of mutated bases in OR/ORF5-2 reached 81% (Fig 2C). There were 245, 198, 105, and 129 singleton variable sites in populations T127, A58, B35, and J34, respectively and 450, 332, 315, and 296 parsimony-informative sites in populations T127, A58, B35, and J34, respectively (Fig 2D). Venn diagrams showed that 240 parsimony-information sites were present in the four populations T127, A58, B35, and J34 simultaneously, but none of singleton variable sites were present in the four populations T127, A58, B35, and J34 simultaneously (Fig 2D).

At least three conserved domains were detected in the 127 sequence, and were concentrated in 1–144 bp, 2398–2832 bp, and 3001–3164 bp regions with lower nucleotide sequence diversities. The second domain (2398–2832 bp) was identified in the overlapping region of ORF5-1 (16–2829 bp) and ORF5-2 (2462–3073 bp), whereas the first domain (1–144 bp) and the third domain (3001–3164 bp) were identified in the 5’UTR and 3’UTR respectively (Fig 2E). Three structural domains (nt 2407–2468, nt 2503–2570, and nt 2618–2739) were predicted in the nucleotide sequence of overlapping regions, and these domains contained the regions of lowest nucleotide sequence diversity (Fig 2F).

The nucleotide diversity of ORF5-1 (π = 0.039) was greater than that of ORF5-2 (π = 0.027), though this difference was not statistically significant (*P* > 0.05). The nucleotide diversity of both ORF5-1 (π = 0.039) and ORF5-2 (π = 0.027) were detectably higher than that of OR (π = 0.011) (*P* < 0.05) (Fig 2G). Nucleotide sequence diversity did not differ across the four subgroups either in the S5 full-length sequence region or in the overlapping sequence region (*P* > 0.05) (Fig 2H).

### Amino acid sequence variation and codon usage factors analysis

**Fig 3:**
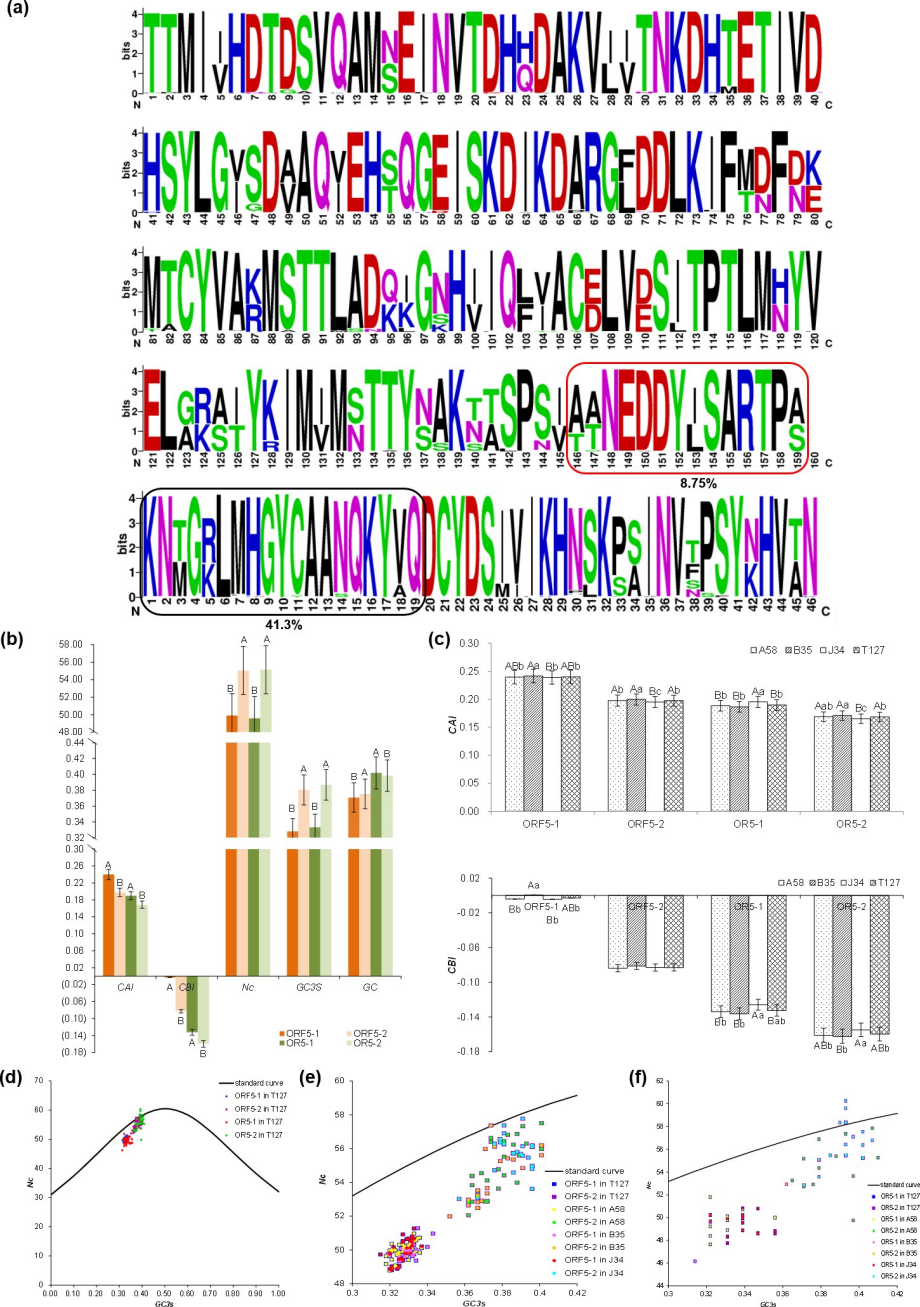
Analyses of amino acid mutation and codon bias use for S5. (**a):** Logo for amino acid mutation coded by ORF5-1 and ORF5-2. **(b):** Significance analysis of the *CAI*, *CBI*, *Nc*, *GC3s*, and *GC* values in T127. (**c):** Significance analysis among the four groups of ORF5-1, ORF5-2, OR5-1, and OR5-2. (**d):** *Nc*-plot of ORF5-1, ORF5-2, OR5-1, and OR5-2 in T127. (**e):** *Nc*-plot of ORF5-1 and ORF5-2 in four subpopulations. (**f):** *Nc*-plot of OR5-1 and OR5-2 in four subpopulations.

ORF5-1 contains 2841 nucleotides and translates 938 amino acids, while ORF5-2 contains 612 nucleotides and translates 204 amino acids. A total of 160 amino acid changes were detected in ORF5-1, with an average of one mutation site per six amino acids. 46 amino acid changes were detected in ORF5-2, with an average of one mutation site per four amino acids. Interestingly, ORF5-1 partially overlapped ORF5-2, but in a different reading frame. 14 amino acid mutation sites were detected within OR5-1 (amino acids of ORF5-1 in overlapping region, nt 2464–2826) with 121 amino acids, which accounted for 8.75% of the total amino acid variation in ORF5-1(Fig 3A). 19 amino acid mutation sites were detected in OR5-2 (amino acids of ORF5-2 in overlapping region, nt 2462 to 2827) with 122 amino acids, which accounted for 41.3% of the total amino acid variation in ORF5-2 (Fig 3A). In OR5-1, the amino acid mutation sites in A58, B35, and J34 respectively accounted for 11.11%, 5.75% and 6.98% of the total variation on ORF5-1, while these mutation sites accounted for 40.63%, 17.39% and 42.86% of the total variation on ORF5-2. This suggests that this overlapping segment may play a special role in the processes of RBSDV S5 genetics and evolution.

In 127 isolates, the *CAI* and *CBI* values of ORF5-1 were significantly higher than those of ORF5-2 (*P* < 0.01). The *CBI* value of ORF5-1 was close to zero, while the *CBI* values of ORF5-2, OR5-1, and OR5-2 were negative, indicating that ORF5-1 has an absolute advantage over ORF5-2 in gene expression and superior codon usage (Fig 3B). The *GC* content of overlapping regions accounted for 13.58% of the total *GC* content of ORF5-1 and 63.50% of the total *GC* content of ORF5-2, indicating that the overlapping region may have a unique effect on the activation mechanism of ORF5-2 (Fig 3B).

The *CAI*, *CBI*, *Nc*, *GC3s*, and *GC* values varied significantly between four populations (A58, B35, J34, and T127) (*P* < 0.01). The *CAI* and *CBI* values in ORF5-1 and ORF5-2 for B35 were significantly higher than those of J34, and the *CBI* value was only positive in ORF5-1, which indicated that gene expression of B35 is higher than that of J34 (Fig 3C). The *CAI* and *CBI* values in OR5-1 for B35 were substantially higher than those of J34, but the opposite pattern was observed in OR5-2, indicating that ORF5-2 expression for B35 depends on OR5-2, but is not consistent with expression in ORF5-1 (Fig 3C). *CAI*, *CBI*, *Nc*, *GC3s* and *GC* values did not differ detectably between T127 and A58, indicating A58 could represent the overall situation of 127 isolates on the codon bias (Fig 3B and 3C).

The *Nc* and *GC3s* were significantly higher for ORF5-2 than for ORF5-1 (*P* < 0.01), and these results are consistent with those observed with OR5-1 and OR5-2 (*P* < 0.01) (Fig 3B). The distribution of points above the standard curve for ORF5-2 and OR5-2, but not for ORF5-1 and OR5-1, suggest that ORF5-1 and OR5-1 have additional independent codon usage bias (Fig 3D). Most of the points for ORF5-1 are far more distant from the standard curve than those of ORF5-2 among the four groups, indicating that the codon usage bias of ORF5-1 is mainly affected by the selection pressure and that ORF5-2 is primarily influenced by mutation pressure (Fig 3E). The mutation pressure of ORF5-2 is obvious in B35 but not obvious in OR5-2 in the overlapping region, while the mutation pressure of OR5-2 in J34 is more prominent (Fig 3F).

### Recombination and phylogenetic analysis of RBSDV isolates

A total of eight recombination events, including four isolates from J34, two isolates from A58, and two isolates from B35, were detected within S5 from 127 isolates, the parents of which were seven isolates from J34, four isolates from A58 and five isolates from B35. Breakpoint positions within the major and minor parental sequences were nt 2075 in ORF 5–1 and nt 3088 in the 3’ UTR of 14NM23 for isolates 14BM7 and 14VIIIM-1. Two parents of the recombinant events, 14BM20 and 14NM17, were not affected by the years, hosts and geographic locations, but their beginning breakpoints were located in 3'UTR and ending breakpoints were located in ORF5-1, which indicated that reorganization in isolate 14BM20 and 14NM17 occurred randomly. The recombinant fragments from the parents in the recombinant events 14NM23, 14BM20, and 14NM17 were across the overlapping region, indicating that the overlapping region is preserved in natural selection because of its special role (Table 1).

**Table 1:** Analysis of possible recombination events in S5 sequences, detected using various algorithms.

Recombinant Sequence	Major parent	Minor parent	Detection methods							Beginning breakpoint	Ending breakpoint
			R	G	B	M	C	S	T		
14BM20	14BM18	14BM22	5.131×10^−3^	5.437×10^−3^	2.120×10^−6^	1.445×10^−8^	2.153×10^−5^	3.3821×10^−19^	1.874×10^−27^	3087	2064
14NM43	14ⅢM-2	14NM14	3.360×10^−20^	2.474×10^−19^	1.348×10^−15^	8.928×10^−15^	3.048×10^−15^	7.729×10^−18^	4.179×10^−35^	1184	2064
14ⅢM-1	14ⅢM-2	14BM18	4.334×10^−9^	1.196×10^−9^	-	1.489×10^−12^	9.169×10^−13^	5.624×10^−16^	1.084×10^−19^	1201	2032
14NM23	14BM7	14VⅢM-1	4.719×10^−15^	1.277×10^−13^	-	7.717×10^−11^	6.531×10^−11^	8.168×10^−11^	1.313×10^−21^	2075	3088
14NM17	14NM25	14NM9	4.429×10^−13^	1.342×10^−13^	4.634×10^−13^	7.217×10^−13^	2.467×10^−13^	3.346×10^−12^	7.782×10^−24^	3089	1293
14BM2	14BM18	13VR-5	7.013×10^−4^	4.212×10^−2^	3.373×10^−3^	6.521×10^−7^	-	5.293×10^−11^	6.413×10^−7^	191	2010
14NM25	14NM28	14NM26	1.404×10^−4^	5.479×10^−4^	6.209×10^−3^	5.452×10^−6^	5.111×10^−6^	8.996×10^−7^	6.096×10^−9^	1207	2076
14VⅢR-1	14NM28	14NM21	9.560×10^−4^	1.440×10^−2^	-	3.329×10^−2^	2.964×10^−2^	1.257×10^−3^	1.468×10^−5^	1265	1883

The abbreviations R, G, B, M, C, S and T represent the algorithms in RDP software including RDP, Geneconv, Bootscan, Maxchi, Chimaera, Siscan and 3seq, respectively.

**Fig 4:**
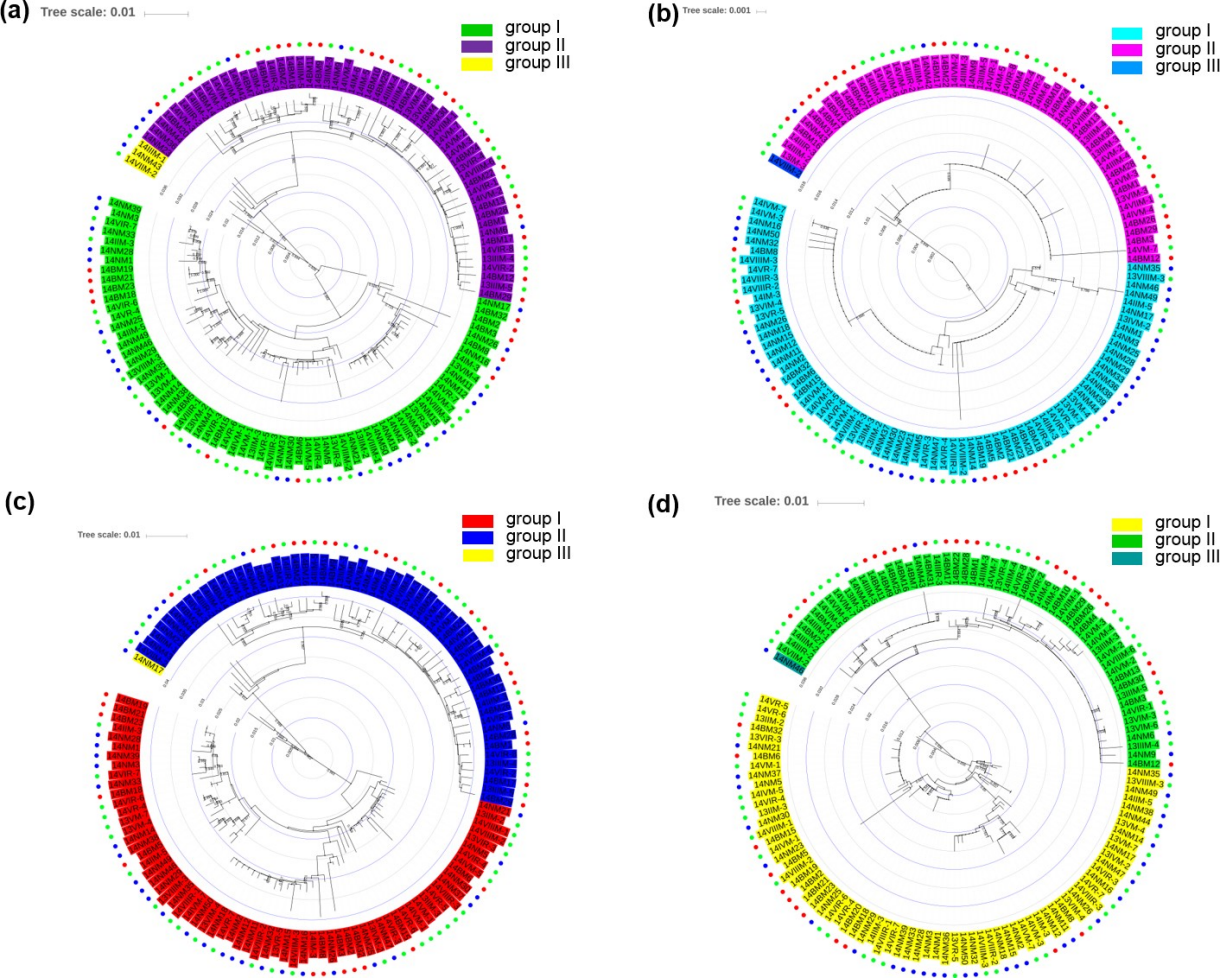
Phylogenetic tree of segment 5 for 127 isolations based on S5 sequences (**a)**, OR sequences (**b)**, ORF5-1 sequences (**c)**, and ORF5-2 sequences (**d)** by neighbor-joining approach. The green, red, and blue circles respectively represent subgroups A58, B35, and J34.

To determine the evolutionary relationships among A58, B35, and J34, a neighbor-joining tree was constructed for the RBSDV S5 sequences. The 127 isolates were classified into three main groups based on S5 sequence designated I, II, and III (Fig 4A), which was consistent with evolutionary result of these sequences based on ORF5-1, ORF5-2, and OR (Fig 4B, 4C and 4D). Group II had few fractionated isolates, though the isolates for B35 were mainly assigned to group III, and the isolates for J34 were mainly assigned to group I, while isolates for A58 were evenly distributed into group I and group III. Group II was not composed of B35 isolates. Interestingly, the evolutionary composition of S5, ORF5-1, ORF5-2, and OR was essentially the same, indicating that the overlapping region could be evolutionarily representative of the whole segment 5.

### Selection pressure and population expansion

**Fig 5:**
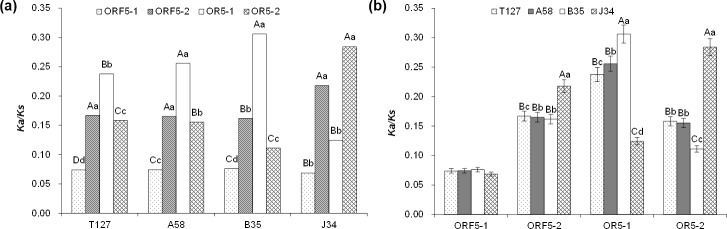
Significance analysis among ORF5-1, ORF5-2, OR5-1, and OR5-2 (P < 0.01) (**a)** and four subpopulations (P < 0.01) (**b)** of selection pressure for S5.

Selection pressure differences across T127, A58, B35, and J34 were quantified with the ratio of non-synonymous to synonymous substitutions (*Ka/Ks*). *Ka/Ks* ratios for ORF5-1, ORF5-2, OR5-1, and OR5-2 indicated negative, or purifying, selection. *Ka/Ks* ratios for ORF5-2 in four groups (A58, B35, J34, and T127) were substantially less negative than those for ORF5-1 (*P* < 0.01), suggesting that selection pressure was greater on ORF5-1 than on ORF5-2 (Fig 5A). For T127, A58, and B35, the *Ka/Ks* ratio in OR5-1 was higher than in OR5-2 (*P* < 0.01) but was lower than in OR5-2 in J34 (*P* < 0.01), which indicated the selection pressure was different between B35 and J34 in the overlapping region (Fig 5A).

The difference between T127, A58, B35, and J34 for *Ka/Ks* ratios of ORF5-1 were not detectably different, indicating selection pressures were similar across the sampled populations for ORF5-1. In ORF5-2, the *Ka/Ks* ratio of J34 was the highest and its selection pressure was lowest (*P* < 0.01) (Fig 5B). In OR5-1, the selection pressure of B35 was lower than A58 and the selection pressure of J34 was higher than A58 (p<0.01, whereas the opposite selection pressure pattern was observed in OR5-2) (Fig 5B). These results suggested no difference between A58 and T127, and that A58 could represent the whole population of T127. The subpopulations B35 and J34 represent two levels of extremes.

Tajima’s D, Fu and Li’s D, and Fu and Li’s F showed negative values for the subpopulations of T127, A58, and J34 but not for B35. The *P* values for Fu and Li’s D and F for T127 were less than 0.05 in ORF5-1 and less than 0.01 in ORF5-2, OR5-1, and OR5-2 (Table 2). This finding suggests that the RBSDV populations were experiencing expansion in T127, especially in the OR. The subpopulations of B35 were also expanding in ORF5-1 and ORF5-2, though not significantly and were in a neutral test or contraction state in OR5-1 and OR5-2 (Table 2).

**Table 2:** Neutrality tests and haplotypes of S5 in the subpopulations.

Subpopulation		Tajima's D	Fu and Li's D	Fu and Li's F	Haplotype number	Haplotype diversity
**ORF5-1**	**T127**	-0.50712	-3.04840*	-2.24006	121	0.99900±0.00000
	**A58**	-0.10332	-1.90787	-1.43449	57	0.99900±0.00001
	**B35**	0.19091	-0.09047	0.00962	31	0.99200±0.00009
	**J34**	-0.34840	-0.46349	-0.50323	33	0.99800±0.00006
**ORF5-2**	**T127**	-0.72555	-3.95700**	-3.03447**	77	0.98000±0.00003
	**A58**	-0.29103	-3.03729	-1.63926	42	0.97900±0.00010
	**B35**	0.40438	-0.00026	0.16326	25	0.97100±0.00026
	**J34**	-1.04258	-0.100282	-1.20464	23	0.96300±0.00034
**OR5-1**	**T127**	-0.66670	-3.72278**	-2.99249**	28	0.86900±0.00021
	**A58**	-0.24841	-2.26154	-1.83418	19	0.88800±0.00050
	**B35**	1.27571	0.39270	0.78862	7	0.77000±0.00223
	**J34**	-1.17592	-0.96547	-1.21875	15	0.87500±0.00153
**OR5-2**	**T127**	-0.73148	-3.90783**	-3.75216**	29	0.87200±0.00021
	**A58**	-0.34709	-2.44470*	-2.01417	20	0.89200±0.00051
	**B35**	1.27571	0.39270	0.78862	7	0.77000±0.00223
	**J34**	-1.17592	-0.96547	-1.21875	15	0.87500±0.00153

*, *P* < 0.05

**, *P* < 0.01

The average values of haplotype diversity were 0.997 in ORF5-1 and 0.973 in ORF5-2, and 0.851 in OR5-1 and 0.852 in OR5-2 (Table 2). This result supports the finding above of RBSDV population expansion and shows haplotype diversity was drawn from mainly from the overlapping area of ORF5-1 and ORF5-2.

### Genetic differentiation and gene flow between subpopulations

The Ks*, Z, and Snn indicated that there was not significant differentiation between subpopulations defined from T127 or A58 (*P* > 0.05) (Table 3). A58 could represent the genetic differentiation of all isolates from RBSDV S5 (Table 3). The genetic differentiation of subpopulations derived from the combinations of T127-B35, T127-J34, A58-B35, A58-J34, and B35-J34 reached significant or extremely significant levels (Table 3).

**Table 3:** Genetic differentiation and gene flow of S5 in the subpopulations.

Subpopulations	Ks* (P-value)	Z (P-value)	Snn (P-value)	*F*_*ST*_	*Nm*
**T127-A58**	5.52516(1.000)	8538.06161(0.866)	0.29685(1.000)	-0.08910	-28.32
**T127-B35**	4.50866(0.071)	6435.59035(0.026)*	0.55545(0.996)	0.04270	5.61
**T127-J34**	4.50571(0.005)**	6255.54551(0.001)**	0.54503(1.000)	0.08200	2.80
**A58-B35**	4.45682(0.057)	2115.92114(0.099)	0.66846(0.006)**	0.02930	8.27
**A58-J34**	4.45097(0.003)**	1974.06965(0.002)**	0.69928(0.001)**	0.10510	2.13
**B35-J34**	4.38461(0.000)***	1001.22113(0.000)***	0.76812(0.000)***	0.24200	0.78

*, 0.01 < *P* < 0.05

**, 0.001 < *P* < 0.01

***, *P* < 0.001

The combined groups of T127-A58, T127-B35, T127-J34, A58-B35, and A58-J34 had absolute *Fst* values less than 0.33 and absolute *Nm* values higher than 1, indicating frequent gene flow occurrence but lower genetic differentiation between these RBSDV subpopulations (Table 3). However, the combined group of B35-J34 showed absolute *Fst* values less than 0.33 and absolute *Nm* values less than 1, indicating not only frequent gene flow occurrence but more genetic differentiation between B35-J34. The subpopulations of B35 and J34 were differentiated to two extremes (Table 3). The combined group of A58-B35 had the smallest absolute *Fst* values, indicating that these two subpopulations experienced the most frequent gene flow (Table 3).

### Interaction screening between P5-1 or P5-2 and other RBSDV proteins by two-hybrid screening

**Fig 6:**
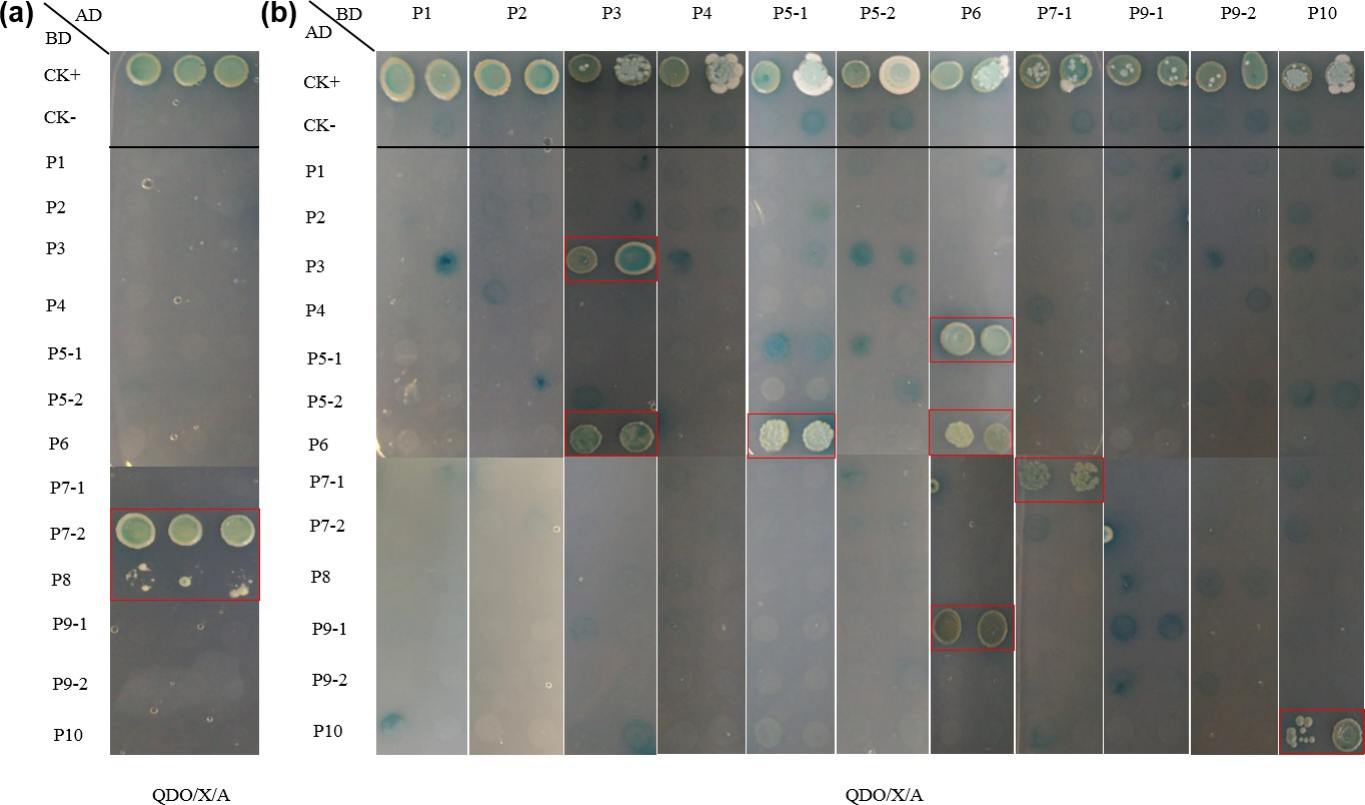
Autoactivation (**a)** and interaction verification (**b)** of RBSDV encoded proteins by two-hybrid screening assays.

To further confirm the mode of pairwise interaction between two encoded S5 proteins and other proteins of RBSDV, yeast two-hybrid screening tests were performed. First, we investigated the interactions between 13 RBSDV-coded proteins, and found that RBSDV P7-2 and P8 act as transcription activation domains in this system and both of them are therefore unsuitable for two-hybrid screening analyses (Fig 6A). The analysis of all possible 143 combinations revealed that P5-1 has an interaction with P6 but no interaction with other RBSDV proteins. No interaction was identified between P5-2 and other RBSDV proteins (Fig 6B). In addition, we identified interactions between structural protein P3 and non-structural protein P6, and between non-structural protein P6 and non-structural protein P9-1. The self-interactions of P3, P6, P7-1 and P10 were also detected (Fig 6B).

## Discussion

RBSDV is a global pathogenic plant virus, which can cause significant losses in maize yield, especially in the Yellow and Huai River valleys of China [31–33]. Previous studies of RBSDV have reported on genetic structure and variability across host plant species or population, time, and location [21,34,35]. However, variation of the two ORFs of RBSDV S5 had not been previously described. We found that the mutations sites in nucleic acids and amino acids of ORF5-2 were much greater than those of ORF5-1, especially the ratio OR/ORF5-2. Codon bias analysis indicated that ORF5-2 and OR5-2 had higher *Nc* values and lower *CAI* values compared with ORF5-1 and OR5-1, and negative *CBI* values which were associated with the use of rare codons. These results all indicated that ORF5-2 was under the greatest selection pressure, continuously evolving, driving variable viral functions, which was consistent with our previous findings [3]. Previous reports have shown that many factors could affect codon usage in viruses, including mutational bias [36], translational selection [37], protein secondary structure [38,39], genomic architecture [40], replicational and transcriptional selection [41], energy efficiency trade-offs [42], and environmental factors [43]. The energy efficiency trade-offs showed that the *GC* content of the genome determines the distribution of cellular energy between RNA and protein, while the low *GC*% of the genome does not afford many coding gene [42]. We found that the *GC* content of overlapping regions accounted for 13.58% of the total *GC* content of ORF5-1 and 63.50% in ORF5-2, indicating that ORF5-2 may have an indispensable role in the viral genome. The translation of RBSDV ORF5-2 may be through a re-initiation mechanism or the internal ribosome entry model [44], and this mechanism requires further investigation.

Recombination allows increased genetic diversity and subsequent adaptation to novel hosts and habitats for RNA viruses [21, 45], and can drive plant virus evolution [46,47]. Recombination has been inferred or observed in the RBSDV segments but not in S5 [21,34,35]. Most of the recombinant fragments are from overlapping fragments or 1kb before overlapping fragments. Half of the eight reorganization events came from subgroup J34, indicating that subgroup J34 is adapting in response to environmental pressures. Previous results have shown that the probability of recombination may be dependent on the size or characteristics of a given RBSDV segment, which was confirmed in our studies. RBSDV S5 has interspecific and intraspecific genetic recombination, suggesting that genetic recombination may be one of the main drivers of RBSDV evolution.

In order to explore the evolution of population genetic structure of RBSDV and the molecular mechanism of population genetic variation, we divided 127 RBSDV sample isolates into 3 subgroups for further study. In our study, the subpopulation of B35 and J34 fell into two extremes. Subgroups B35 and J34 evolved into two branches due to different selection pressures especially in the overlapping region between them. Gene flow is an important factor that drive evolution of RBSDV in China based on subpopulations from S8 with 101 samples and S10 with 103 samples [34]. Although gene flow occurred frequently between subgroups B35 and J34, the genetic differentiation was more striking. Interestingly, subgroup A58 is similar to the total population T127 in terms of the RBSDV evolution of population genetic structure. Studies have shown that disease outbreaks, geographic origin, host range, and mediators are closely related to the genetic diversity and structure plant viruses. Population structure and genetic diversity of rice tungro spherical virus (RTSV) are significantly higher in disease-prone areas than in the outbreak region [48]. Therefore, the different population structure of subpopulation J34 from Jining (Shandong Province) and subpopulation B35 from Beijing may be the cause of the epidemic of MRDD observed in Shandong Province.

RNA viruses which infected plants or other taxa have many strategies to express their genomes, including multi-partite genomes, sub-genomic RNAs, translational frameshifting, overlapping reading frames, and changes to replication strategies [10,14]. This adaptability and wide diversity allow plant viruses to colonize many host species. The number of genomic segments is limited by the recycling replicase complex of toxic particles, therefore many of the ds-RNA viruses have adapted to produce variable protein products from a single mRNA source [10]. The presence of the second ORF of RBSDV S5 may also be limited by the number of genomic fragments. In this study, we investigated interactions between 13 RBSDV proteins by yeast two-hybrid screening assays and identified interactions of P5-1/P6, P6/P5-1, P6/P6, and P6/P9-1, which was consistent with the results of previous studies [2,5,49]. RBSDV P6 encoded for a protein RNA silencing suppressor, which significantly inhibits DNA methylation of the plant genome [50]. Therefore, a large number of viral matrix components are likely needed to enhance the virus itself when RBSDV P6 competes with the host factors. The strong interaction between P5-1 and P6 may act as a signal to activate transcriptional expression of P5-2, which was demonstrated to be located in the chloroplasts in RBSDV-infected plant cells [6]. RBSDV S3 was previously reported to code for a protein guanylyltransferase, which was similar to the function of mycorevirus-1 VP3 [51]. In this study, we investigated interactions of P3/P3 and P3/P6, and we hypothesized that RBSDV P3 acts as a cofactor assisting RBSDV P6 in inhibiting host genomic DNA methylation. In our study, RBSDV P7-1 self-interaction was also detected, which led us to speculate that P7-1 acts as a bridge to help the virus shuttle between adjacent cells. Above all, RBSDV P5-1 and P5-2, structural and non-structural proteins, respectively, did not function like the RBSDV S7 and S9 encoded proteins. In future research, we will study the interaction and recognition modes between RBSDV encoded proteins.

## Supporting information

S1 Table Information of RBSDV S5 sequences. (DOCX)

S2 Table Specific primers used for amplifying and sequencing S5 sequences. (DOCX)

S3 Table Infusion primers used for amplifying RBSDV sequences. (DOCX)
