# Supplementary material for: Molecular characteristics of segment 5, a unique fragment encoding two partially overlapping ORFs in the genome of rice black-streaked dwarf virus
Source: PLoS One. 2019 Nov 7;14(11):e0224569. doi: 10.1371/journal.pone.0224569 (PMC6837423; doi:10.1371/journal.pone.0224569)
Supplement: S1 Table — (DOCX) [file pone.0224569.s001.docx]

**S1 Table. Information of RBSDV S5 sequences.**

| Subpopulation | Code | Location (City, Province) | Host | Latitude | Longitude |
| --- | --- | --- | --- | --- | --- |
| A58 | 13IM-3 | Beijing | maize | 39°57′ | 116°19′ |
| A58 | 13IIM-2 | Tangshan, Hebei | maize | 39°37′ | 118°10′ |
| A58 | 13IIM-3 | Tangshan, Hebei | maize | 39°37′ | 118°10′ |
| A58 | 13IIIM-3 | Baoding, Hebei | maize | 38°52′ | 116°02′ |
| A58 | 13IIIM-4 | Baoding, Hebei | maize | 38°52′ | 116°02′ |
| A58 | 13IIIM-5 | Baoding, Hebei | maize | 38°52′ | 116°02′ |
| A58 | 13IVM-2 | Jinan, Shandong | maize | 36°39′ | 117°06′ |
| A58 | 13VM-4 | Jining, Shandong | maize | 35°24′ | 116°34′ |
| A58 | 13VM-7 | Jining, Shandong | maize | 35°24′ | 116°34′ |
| A58 | 13VR-5 | Jining, Shandong | Rice | 35°24′ | 116°34′ |
| A58 | 13VIM-3 | Zhengzhou, Henan | maize | 34°43′ | 113°37′ |
| A58 | 13VIM-4 | Zhengzhou, Henan | maize | 34°43′ | 113°37′ |
| A58 | 13VIM-6 | Zhengzhou, Henan | maize | 34°43′ | 113°37′ |
| A58 | 13VIR-3 | Zhengzhou, Henan | Rice | 34°43′ | 113°37′ |
| A58 | 13VIR-4 | Zhengzhou, Henan | Rice | 34°43′ | 113°37′ |
| A58 | 13VIIIM-3 | Nanjing, Jiangsu | maize | 32°02′ | 118°52′ |
| A58 | 14IIM-3 | Tangshan, Hebei | maize | 39°36′ | 118°55′ |
| A58 | 14IIM-5 | Tangshan, Hebei | maize | 39°36′ | 118°55′ |
| A58 | 14IIIM-1 | Baoding, Hebei | maize | 38°52′ | 116°02′ |
| A58 | 14IIIM-2 | Baoding, Hebei | maize | 38°52′ | 116°02′ |
| A58 | 14IIIM-3 | Baoding, Hebei | maize | 38°52′ | 116°02′ |
| A58 | 14IIIM-4 | Baoding, Hebei | maize | 38°52′ | 116°02′ |
| A58 | 14IIIM-5 | Baoding, Hebei | maize | 38°52′ | 116°02′ |
| A58 | 14IIIR-2 | Baoding, Hebei | Rice | 38°52′ | 116°02′ |
| A58 | 14IIIR-3 | Baoding, Hebei | Rice | 38°52′ | 116°02′ |
| A58 | 14IVM-1 | Jinan, Shandong | maize | 36°39′ | 117°06′ |
| A58 | 14IVM-2 | Jinan, Shandong | maize | 36°39′ | 117°06′ |
| A58 | 14IVM-3 | Jinan, Shandong | maize | 36°39′ | 117°06′ |
| A58 | 14IVM-4 | Jinan, Shandong | maize | 36°39′ | 117°06′ |
| A58 | 14IVM-5 | Jinan, Shandong | maize | 36°39′ | 117°06′ |
| A58 | 14IVM-7 | Jinan, Shandong | maize | 36°39′ | 117°06′ |
| A58 | 14VM-1 | Jining, Shandong | maize | 35°24′ | 116°34′ |
| A58 | 14VM-2 | Jining, Shandong | maize | 35°24′ | 116°34′ |
| A58 | 14VM-3 | Jining, Shandong | maize | 35°24′ | 116°34′ |
| A58 | 14VM-7 | Jining, Shandong | maize | 35°24′ | 116°34′ |
| A58 | 14VR-4 | Jining, Shandong | Rice | 35°24′ | 116°34′ |
| A58 | 14VR-5 | Jining, Shandong | Rice | 35°24′ | 116°34′ |
| A58 | 14VR-6 | Jining, Shandong | Rice | 35°24′ | 116°34′ |
| A58 | 14VR-7 | Jining, Shandong | Rice | 35°24′ | 116°34′ |
| A58 | 14VIM-5 | Zhengzhou, Henan | maize | 34°43′ | 113°37′ |
| A58 | 14VIM-6 | Zhengzhou, Henan | maize | 34°43′ | 113°37′ |
| A58 | 14VIM-7 | Zhengzhou, Henan | maize | 34°43′ | 113°37′ |
| A58 | 14VIR-1 | Zhengzhou, Henan | Rice | 34°43′ | 113°37′ |
| A58 | 14VIR-2 | Zhengzhou, Henan | Rice | 34°43′ | 113°37′ |
| A58 | 14VIR-3 | Zhengzhou, Henan | Rice | 34°43′ | 113°37′ |
| A58 | 14VIR-4 | Zhengzhou, Henan | Rice | 34°43′ | 113°37′ |
| A58 | 14VIR-6 | Zhengzhou, Henan | Rice | 34°43′ | 113°37′ |
| A58 | 14VIR-7 | Zhengzhou, Henan | Rice | 34°43′ | 113°37′ |
| A58 | 14VIR-8 | Zhengzhou, Henan | Rice | 34°43′ | 113°37′ |
| A58 | 14VIIM-1 | Yancheng, Jiangsu | maize | 33°20′ | 120°09′ |
| A58 | 14VIIM-2 | Yancheng, Jiangsu | maize | 33°20′ | 120°09′ |
| A58 | 14VIIIM-1 | Nanjing, Jiangsu | maize | 32°02′ | 118°52′ |
| A58 | 14VIIIM-2 | Nanjing, Jiangsu | maize | 32°02′ | 118°52′ |
| A58 | 14VIIIM-3 | Nanjing, Jiangsu | maize | 32°02′ | 118°52′ |
| A58 | 14VIIIM-6 | Nanjing, Jiangsu | maize | 32°02′ | 118°52′ |
| A58 | 14VIIIR-1 | Nanjing, Jiangsu | Rice | 32°02′ | 118°52′ |
| A58 | 14VIIIR-2 | Nanjing, Jiangsu | Rice | 32°02′ | 118°52′ |
| A58 | 14VIIIR-3 | Nanjing, Jiangsu | Rice | 32°02′ | 118°52′ |
| B35 | 14IM-3 | Beijing | maize | 39°57′ | 116°19′ |
| B35 | 14IM-5 | Beijing | maize | 39°57′ | 116°19′ |
| B35 | 14IM-6 | Beijing | maize | 39°57′ | 116°19′ |
| B35 | 14BM1 | Beijing | maize | 39°57′ | 116°19′ |
| B35 | 14BM2 | Beijing | maize | 39°57′ | 116°19′ |
| B35 | 14BM3 | Beijing | maize | 39°57′ | 116°19′ |
| B35 | 14BM4 | Beijing | maize | 39°57′ | 116°19′ |
| B35 | 14BM5 | Beijing | maize | 39°57′ | 116°19′ |
| B35 | 14BM6 | Beijing | maize | 39°57′ | 116°19′ |
| B35 | 14BM7 | Beijing | maize | 39°57′ | 116°19′ |
| B35 | 14BM8 | Beijing | maize | 39°57′ | 116°19′ |
| B35 | 14BM9 | Beijing | maize | 39°57′ | 116°19′ |
| B35 | 14BM10 | Beijing | maize | 39°57′ | 116°19′ |
| B35 | 14BM11 | Beijing | maize | 39°57′ | 116°19′ |
| B35 | 14BM12 | Beijing | maize | 39°57′ | 116°19′ |
| B35 | 14BM13 | Beijing | maize | 39°57′ | 116°19′ |
| B35 | 14BM14 | Beijing | maize | 39°57′ | 116°19′ |
| B35 | 14BM15 | Beijing | maize | 39°57′ | 116°19′ |
| B35 | 14BM16 | Beijing | maize | 39°57′ | 116°19′ |
| B35 | 14BM17 | Beijing | maize | 39°57′ | 116°19′ |
| B35 | 14BM18 | Beijing | maize | 39°57′ | 116°19′ |
| B35 | 14BM19 | Beijing | maize | 39°57′ | 116°19′ |
| B35 | 14BM20 | Beijing | maize | 39°57′ | 116°19′ |
| B35 | 14BM21 | Beijing | maize | 39°57′ | 116°19′ |
| B35 | 14BM22 | Beijing | maize | 39°57′ | 116°19′ |
| B35 | 14BM23 | Beijing | maize | 39°57′ | 116°19′ |
| B35 | 14BM24 | Beijing | maize | 39°57′ | 116°19′ |
| B35 | 14BM25 | Beijing | maize | 39°57′ | 116°19′ |
| B35 | 14BM26 | Beijing | maize | 39°57′ | 116°19′ |
| B35 | 14BM27 | Beijing | maize | 39°57′ | 116°19′ |
| B35 | 14BM28 | Beijing | maize | 39°57′ | 116°19′ |
| B35 | 14BM29 | Beijing | maize | 39°57′ | 116°19′ |
| B35 | 14BM30 | Beijing | maize | 39°57′ | 116°19′ |
| B35 | 14BM31 | Beijing | maize | 39°57′ | 116°19′ |
| B35 | 14BM32 | Beijing | maize | 39°57′ | 116°19′ |
| J34 | 14NM1 | Jining, Shandong | maize | 35°24′ | 116°34′ |
| J34 | 14NM2 | Jining, Shandong | maize | 35°24′ | 116°34′ |
| J34 | 14NM3 | Jining, Shandong | maize | 35°24′ | 116°34′ |
| J34 | 14NM5 | Jining, Shandong | maize | 35°24′ | 116°34′ |
| J34 | 14NM6 | Jining, Shandong | maize | 35°24′ | 116°34′ |
| J34 | 14NM9 | Jining, Shandong | maize | 35°24′ | 116°34′ |
| J34 | 14NM11 | Jining, Shandong | maize | 35°24′ | 116°34′ |
| J34 | 14NM12 | Jining, Shandong | maize | 35°24′ | 116°34′ |
| J34 | 14NM14 | Jining, Shandong | maize | 35°24′ | 116°34′ |
| J34 | 14NM15 | Jining, Shandong | maize | 35°24′ | 116°34′ |
| J34 | 14NM16 | Jining, Shandong | maize | 35°24′ | 116°34′ |
| J34 | 14NM17 | Jining, Shandong | maize | 35°24′ | 116°34′ |
| J34 | 14NM18 | Jining, Shandong | maize | 35°24′ | 116°34′ |
| J34 | 14NM21 | Jining, Shandong | maize | 35°24′ | 116°34′ |
| J34 | 14NM23 | Jining, Shandong | maize | 35°24′ | 116°34′ |
| J34 | 14NM25 | Jining, Shandong | maize | 35°24′ | 116°34′ |
| J34 | 14NM26 | Jining, Shandong | maize | 35°24′ | 116°34′ |
| J34 | 14NM28 | Jining, Shandong | maize | 35°24′ | 116°34′ |
| J34 | 14NM29 | Jining, Shandong | maize | 35°24′ | 116°34′ |
| J34 | 14NM30 | Jining, Shandong | maize | 35°24′ | 116°34′ |
| J34 | 14NM32 | Jining, Shandong | maize | 35°24′ | 116°34′ |
| J34 | 14NM33 | Jining, Shandong | maize | 35°24′ | 116°34′ |
| J34 | 14NM35 | Jining, Shandong | maize | 35°24′ | 116°34′ |
| J34 | 14NM36 | Jining, Shandong | maize | 35°24′ | 116°34′ |
| J34 | 14NM37 | Jining, Shandong | maize | 35°24′ | 116°34′ |
| J34 | 14NM38 | Jining, Shandong | maize | 35°24′ | 116°34′ |
| J34 | 14NM39 | Jining, Shandong | maize | 35°24′ | 116°34′ |
| J34 | 14NM42 | Jining, Shandong | maize | 35°24′ | 116°34′ |
| J34 | 14NM43 | Jining, Shandong | maize | 35°24′ | 116°34′ |
| J34 | 14NM44 | Jining, Shandong | maize | 35°24′ | 116°34′ |
| J34 | 14NM46 | Jining, Shandong | maize | 35°24′ | 116°34′ |
| J34 | 14NM47 | Jining, Shandong | maize | 35°24′ | 116°34′ |
| J34 | 14NM49 | Jining, Shandong | maize | 35°24′ | 116°34′ |
| J34 | 14NM50 | Jining, Shandong | maize | 35°24′ | 116°34′ |
