# Supplementary material for: Molecular characteristics of segment 5, a unique fragment encoding two partially overlapping ORFs in the genome of rice black-streaked dwarf virus
Source: PLoS One. 2019 Nov 7;14(11):e0224569. doi: 10.1371/journal.pone.0224569 (PMC6837423; doi:10.1371/journal.pone.0224569)
Supplement: S2 Table — (DOCX) [file pone.0224569.s002.docx]

**S2 Table. Specific primers used for amplifying and sequencing S5 sequences.**

| Primer | Sequence (5’-3’) | S5 nt region |
| --- | --- | --- |
| S5-F-1 | AGTTTTTTTCACTCATGACATATTCG | 1-26 |
| S5-1-1 | TTCCTTCTTTCAGAAACTGATATGG | 1316-1339 |
| S5-3-1 | AG*GC*TTATTGAAAGATGGACTT | 1128-1149 |
| S5-4-1 | CAAACAGCTCATGGTTGAAAAC | 2178-2199 |
| S5-2-1 | AC*GC*AAACCTTATTTCCGATTC | 1842-1864 |
| S5-R-1 | *GC*ATCTAAGGAGACACAGAACCC | 3117-3139 |
