# Supplementary material for: Molecular characteristics of segment 5, a unique fragment encoding two partially overlapping ORFs in the genome of rice black-streaked dwarf virus
Source: PLoS One. 2019 Nov 7;14(11):e0224569. doi: 10.1371/journal.pone.0224569 (PMC6837423; doi:10.1371/journal.pone.0224569)
Supplement: S3 Table — (DOCX) [file pone.0224569.s003.docx]

**S3 Table. Infusion primers used for amplifying RBSDV sequences.**

| Primer | Sequences（5’-3’） |
| --- | --- |
| S1-BD-E-f | GGCCATGGAGGCCGAATTCATGGTGGAACGAAAGTTCAG |
| S1-BD-B-r | GCTGCAGGTCGACGGATCCGTCAGTCGAACTCCAGAG |
| S2-BD-E-f | GGCCATGGAGGCCGAATTCATGAATTCTGAGGAG |
| S2-BD-B-r | GCTGCAGGTCGACGGATCCGTTACAACTGCGATGAC |
| S3-BD-E-f | GGCCATGGAGGCCGAATTCATGTTGAAAGTAAACGTGC |
| S3-BD-B-r | GCTGCAGGTCGACGGATCCGTCATTTCTTTGGGTGAATTAGG |
| S4-BD-E-f | GCCATGGAGGCCGAATTCATGGATCCAGGACAAGTCCTC |
| S4-BD-B-r | TGCAGGTCGACGGATCCGTTAAAATCTCAGAATTTCCGGG |
| S5-1-BD-E-f | GGCCATGGAGGCCGAATTCATGACATATTCGAAAGTGAAG |
| S5-1-BD-B-r | GCAGGTCGACGGATCCGTCACGTTGAAGATGGTTGAA |
| S5-2-BD-E-f | GGCCATGGAGGCCGAATTCATGACAAAATTTCCACTTG |
| S5-2-BD-B-r | TGCAGGTCGACGGATCCGTCAGAGATGAAGCATGACTC |
| S6-BD-E-f | GGCCATGGAGGCCGAATTCATGTCTGCCCACCTG |
| S6-BD-B-r | GCTGCAGGTCGACGGATCCGTTACTCAGAGCTTAGTTG |
| S7-1-BD-E-f | GGCCATGGAGGCCGAATTCATGGATAGACCTGCTCGAGAA |
| S7-1-BD-B-r | GCTGCAGGTCGACGGATCCGTTAAGCAGAAGGAGATGA |
| S7-2-BD-E-f | GGCCATGGAGGCCGAATTCATGAATTACACTTTAAGTGATCATTACGC |
| S7-2-BD-B-r | GCTGCAGGTCGACGGATCCGTTAAGAATTCAGTATCTTTTTGATC |
| S8-BD-E-f | TATGGCCATGGAGGCCGAATTCATGACTGGCACCCATGACGA |
| S8-BD-B-r | GCTGCAGGTCGACGGATCCGTTATACAATAATCGATGAAGC |
| S9-1-BD-E-f | CATGGAGGCCGAATTCATGGCAGACCAAGAGCGGAGAAC |
| S9-1-BD-B-r | GCAGGTCGACGGATCCGTCAAACGTCCAATTTCAAGGAAG |
| S9-2-BD-E-f | GGCCATGGAGGCCGAATTCATGAATCCCCAATCTTCAGTAAATG |
| S9-2-BD-B-r | GCAGGTCGACGGATCCGTTAATTAAAAAGCGTATAGTTTAC |
| S10-BD-E-f | TATGGCCATGGAGGCCGAATTCATGGCTGACATAAGACTCG |
| S10-BD-B-r | GCTGCAGGTCGACGGATCCGTCATCTTGTCACTTTGTTTAATAC |
| S1-AD-E-f | GGAGGCCAGTGAATTCATGGTGGAACGAAAGTTCAG |
| S1-AD-B-r | GCTCGAGCTCGATGGATCCGTCAGTCGAACTCCAGAGTAAG |
| S2-AD-E- f | CATGGAGGCCAGTGAATTCATGAATTCTGAGGAG |
| S2-AD-B-r | TCGAGCTCGATGGATCCGTTACAACTGCGATGAC |
| S3-AD-E-f | CATGGAGGCCAGTGAATTCATGTTGAAAGTAAACGTGC |
| S3-AD-B-r | TCGAGCTCGATGGATCCGTCATTTCTTTGGGTGAATTAGG |
| S4-AD-E-f | CATGGAGGCCAGTGAATTCATGGATCCAGGACAAGTCCTC |
| S4-AD-B-r | TCGAGCTCGATGGATCCGTTAAAATCTCAGAATTTCCGGGTG |
| S5-1-AD-E-f | GGCCATGGAGGCCAGTGAATTCATGACATATTCGAAAGTG |
| S5-1-AD-B-r | TCGAGCTCGATGGATCCGTCACGTTGAAGATGGTTGAAG |
| S5-2-AD-E-f | GGCCATGGAGGCCAGTGAATTCATGACAAAATTTCCACTTG |
| S5-2-AD-B-r | TCGAGCTCGATGGATCCGTCAGAGATGAAGCATGACTCCT |
| S6-AD-E-f | GGAGGCCAGTGAATTCATGTCTGCCCACCTGACC |
| S6-AD-B-r | GCTCGAGCTCGATGGATCCGTTACTCAGAGCTTAGTTGC |
| S7-1-AD-E-f | GGAGGCCAGTGAATTCATGGATAGACCTGCTCGAGAACA |
| S7-1-AD-B-r | GCTCGAGCTCGATGGATCCGTTAAGCAGAAGGAGATGAAAAG |
| S7-2-AD-E-f | GCCATGGAGGCCAGTGAATTCATGAATTACACTTTAAGTGATCATTACGC |
| S7-2-AD-B-r | GCTCGAGCTCGATGGATCCGTTAAGAATTCAGTATCTTTTTGATC |
| S8-AD-E-f | TGGAGGCCAGTGAATTCATGACTGGCACCCATGACGA |
| S8-AD-B-r | GCTCGAGCTCGATGGATCCGTTATACAATAATCGATGAAGC |
| S9-1-AD-E-f | CATGGAGGCCAGTGAATTCATGGCAGACCAAGAGCGGAGAAC |
| S9-1-AD-B-r | TCGAGCTCGATGGATCCGTCAAACGTCCAATTTCAAGGAAG |
| S9-2-AD-E-f | GGCCATGGAGGCCAGTGAATTCATGAATCCCCAATCTTCAGTAAATG |
| S9-2-AD-B-r | GCTCGAGCTCGATGGATCCGTTAATTAAAAAGCGTATAG |
| S10-AD-E-f | TGGAGGCCAGTGAATTCATGGCTGACATAAGACTCG |
| S10-AD-B-r | GCTCGAGCTCGATGGATCCGTCATCTTGTCACTTTGTTTAATAC |
